# Ocular Complication in Facial Aesthetic Laser and Light Treatments: A Comprehensive Review

**DOI:** 10.3390/diagnostics14182006

**Published:** 2024-09-10

**Authors:** Kar Wai Alvin Lee, Lisa Kwin Wah Chan, Angela Wai Kay Lee, Cheuk Hung Lee, Jovian Wan, Kyu-Ho Yi

**Affiliations:** 1 EverKeen Medical Centre, Hong Kong; alvin429@yahoo.com (K.W.A.L.); drchan.everkeen@gmail.com (L.K.W.C.); andylee618@hotmail.com (C.H.L.); 2 The Skin Oracle, Hong Kong; ang3la.l33@gmail.com; 3 Asia Pacific Aesthetic Academy, Hong Kong; 4 Division in Anatomy and Developmental Biology, Department of Oral Biology, Human Identification Research Institute, BK21 FOUR Project, Yonsei University College of Dentistry, 50-1 Yonsei-ro, Seodaemun-gu, Seoul 03722, Republic of Korea; 5 Maylin Clinic (Apgujeong), Seoul 06005, Republic of Korea

**Keywords:** ocular complications, facial esthetics, laser treatments, light therapy, safety protocols, review

## Abstract

Background: The increasing popularity of laser- and light-based esthetic treatments for facial rejuvenation has raised concerns regarding ocular safety. Although these procedures are generally considered safe and effective, there is a growing body of evidence highlighting the potential for ocular complications. This review aims to systematically analyze the types and mechanisms of ocular injuries associated with such treatments, as well as to evaluate preventive measures and management strategies. Methods: A comprehensive literature search was conducted using databases including MEDLINE, PubMed and Ovid for relevant studies published on clinical trials, diagnosis and treatment. Some papers were further reviewed using a double-blinding approach, varying sample sizes, control usage, randomization usage and objective endpoint measurements. All studies were classified according to the Oxford Centre for evidence-based medicine evidence hierarchy. Result: Our review identified several types of ocular complications associated with facial laser or light treatments, including but not limited to conjunctival burns, corneal damage, retinal phototoxicity, and transient vision disturbances. The incidence of these complications varies significantly depending on the type of laser or light source employed, treatment parameters, and the anatomical proximity of the eyes to the treatment area. Factors such as inadequate protective measures, patient movement during the procedure, and the operator’s experience were found to contribute to the risk of ocular injury. Strategies such as the use of appropriate eye protection, careful patient positioning, and thorough pre-treatment assessments were highlighted as essential preventive measures. Conclusion: Ocular complications, though rare, represent a significant risk in facial esthetic laser and light treatments. This review underscores the importance of awareness among practitioners regarding the potential ocular hazards and the implementation of robust safety protocols. Future research is needed to establish standardized guidelines to minimize risks and enhance patient safety in esthetic dermatological practices. Continued education and improved protective strategies will be essential in safeguarding ocular health as the field of esthetic treatments continues to evolve. This comprehensive review serves as an essential resource for practitioners, informing them of ocular risks, management options, and the need for vigilance to mitigate complications in clinical practice.

## 1. Introduction

Due to their diverse and beneficial applications, lasers and light-based therapies have become integral tools in various medical fields. The absorption of light pulse energy by tissues is contingent upon the presence of chromophores such as water, melanin, or hemoglobin and the wavelength of the light used [1,2]. However, the outcomes of these treatments are highly dependent on the power density of the light pulse applied to the tissues. These treatments, offering non-invasive solutions for skin rejuvenation, pigmentation removal, and the treatment of vascular lesions, are becoming increasingly popular [3]. Despite their efficacy, these modalities pose significant risks, particularly concerning ocular health. The spectrum of ocular complications associated with these treatments ranges from mild irritation to severe injuries, including corneal burns, retinal damage, and even permanent vision loss [4].

As these procedures become more widely accessible, it is imperative that practitioners remain acutely aware of the potential for adverse effects, especially given the close anatomical proximity of the eyes to treatment areas [5]. Factors such as inadequate protective measures, the operator’s level of experience, and patient movement during the procedure can significantly increase the risk of ocular damage. Furthermore, the wide variety of laser technologies and treatment protocols available complicates the establishment of standardized safety practices [6].

In esthetic medicine and cosmetology, the most commonly employed lasers include photoablative lasers, which cause rapid tissue vaporization, photothermal (non-ablative) lasers, and lasers that induce photochemical effects for tissue biostimulation [7]. Intense Pulsed Light (IPL) devices are also widely used, leveraging the mechanism of selective photothermolysis to achieve multiple effects on skin tissues [8]. Selecting the appropriate tool, choosing the correct laser type, and thoroughly educating the patient on the procedure are essential steps to ensure optimal therapeutic outcomes.

This review seeks to compile and synthesize the current literature on ocular complications associated with facial esthetic laser and light treatments. By identifying common types of ocular injuries, risk factors, and preventive strategies, this review aims to enhance clinical awareness and promote safer practices in the rapidly evolving field of esthetic dermatology. A summary of key findings from the reviewed studies is provided in Table 1.

## 2. Materials and Methods

The keywords “Ocular complications”, “Periocular complications”, “Laser ocular complications”, “Laser”, and “Intense Pulsed Light” were utilized to search the MEDLINE, PubMed, and Ovid databases for relevant studies focusing on clinical trials, diagnosis, and treatment. Selected papers underwent further evaluation, incorporating criteria such as double-blinding, various sample sizes, control usage, randomization, and objective endpoint measurements. All studies were subsequently classified according to the Oxford Centre for Evidence-Based Medicine’s evidence hierarchy [9].

## 3. Results

### 3.1. Ocular Complications from Various Laser Treatments

Park et al. [10] presented a detailed case report, categorized as Level IV evidence, documenting a severe macular injury caused by a Neodymium-doped Yttrium Aluminum Garnet laser. The patient, who was undergoing a routine laser procedure, suffered a significant macular injury due to a misalignment in the laser system, which led to a marked reduction in visual acuity. This reduction persisted over a six-year follow-up period, during which the patient experienced only partial functional improvement but never returned to baseline levels of visual acuity. The authors emphasized the critical need for rigorous safety protocols in clinical settings to prevent such incidents and discussed the broader implications for laser safety. They also highlighted the importance of proper equipment handling and thorough training of practitioners to minimize the risk of similar injuries.

Chen et al. [11] provided a case report, also classified as Level IV evidence, documenting an incident of retinal injury following a cosmetic laser treatment. In this case, a patient received a cosmetic laser procedure intended for skin rejuvenation but inadvertently suffered retinal damage due to an unintended laser beam exposure. This incident occurred due to a moment of carelessness or misuse during the procedure, which led to severe consequences for the patient’s vision. Following the exposure, the patient presented with symptoms of visual impairment, prompting immediate ophthalmic evaluation. Imaging techniques revealed significant damage to the retinal structure, raising concerns about the potential for long-term visual sequelae. The authors emphasized the necessity for stringent safety protocols in cosmetic laser procedures, particularly given the increasing prevalence of such treatments in non-medical settings. They argued that practitioners must be adequately trained and equipped to prevent such accidents and that stringent operational protocols should be established to reduce the risk of unintended exposure during laser treatments. The implications of this case extend beyond individual safety, prompting discussions about the regulations governing laser use in cosmetic practices.

Widder et al. [12] discussed a case of corneal damage following a carbon dioxide laser skin resurfacing procedure, providing Level IV evidence. The report underscores the potential ocular complications that can arise from cosmetic laser treatments, particularly when there is inadvertent exposure of the eyes to laser light. The patient, who underwent the procedure to improve skin texture and reduce facial wrinkling, presented with symptoms such as ocular discomfort and visual disturbances. A comprehensive ophthalmological examination revealed evidence of corneal injury, including superficial punctate keratitis and corneal opacity. The authors meticulously documented the clinical findings and the management strategies employed to address the ocular injury. They highlighted the importance of immediate identification and treatment of corneal damage to mitigate further complications, such as visual acuity loss or chronic discomfort. The authors also emphasized the significance of providing patients with proper postoperative care instructions, especially regarding eye protection during the recovery phase. A critical aspect of the discussion revolves around the safety measures necessary to prevent such injuries in the future. The authors advocated for stringent protocols, including the routine use of protective eye gear for patients undergoing laser resurfacing and heightened awareness among practitioners regarding the risks associated with laser procedures. They emphasized that while CO_2_ lasers are effective for dermatological purposes, appropriate safeguards are essential to protect the eyes during treatment.

### 3.2. Specific Complications from Periocular Laser Treatments

Hammes et al. [13] reported a case involving pupil damage resulting from laser treatment for a facial port-wine stain, providing additional Level IV evidence. The case focuses on a patient who received laser therapy targeting a port-wine stain located in the periorbital region. Following the treatment, the patient experienced complications characterized by pupillary dysfunction, which included both a direct impact on constriction response and altered pupillary reflexes. This led to significant clinical findings that warranted further evaluation. The authors emphasized the importance of understanding the anatomical proximity of ocular structures to the treatment area when performing laser procedures, especially those involving the face. The potential for collateral damage to nearby ocular components, including the pupil, underscores the necessity of meticulous technique and precautions during treatment. The article discusses the management of the pupil damage, highlighting the steps taken to evaluate and address the complications. The authors also stressed the importance of thorough patient education regarding the risks associated with laser treatments, especially for areas close to the eyes. They advocated for the use of protective measures, such as eye shields, to minimize the risk of ocular injuries during laser procedures. This case contributes to the existing literature by illustrating the potential unintended effects of laser therapy, specifically in sensitive areas such as the face. It serves as a cautionary tale for dermatologists and cosmetic practitioners, emphasizing the need for a comprehensive understanding of the laser technology in use, as well as the importance of taking preventive measures to safeguard patient vision.

Karabela et al. [14] and Gunes et al. [15] both presented cases of anterior uveitis following eyebrow epilation using alexandrite lasers, providing Level IV evidence. In the case reported by Karabela et al., the patient experienced symptoms of eye inflammation, specifically anterior uveitis, shortly following the laser treatment for eyebrow hair removal. The symptoms included redness, pain, and blurred vision, which prompted the patient to seek ophthalmological evaluation. Upon examination, the ophthalmologists confirmed the diagnosis of anterior uveitis and initiated appropriate treatment, which included corticosteroids and mydriatics to reduce inflammation and alleviate pain. The report emphasizes the importance of considering potential ocular complications arising from cosmetic procedures, even those that may seem distal to the eye, such as laser hair removal in the eyebrow region. The authors analyzed the possible mechanisms behind the uveitis development, including direct thermal injury, an inflammatory response to the laser treatment, and the potential for pigment dispersion from the treated area affecting intraocular structures. They underlined the significance of providing patients with adequate pre-treatment information regarding the risks associated with laser procedures in peripheral areas, especially near sensitive anatomical structures like the eyes. This case report serves as a reminder for practitioners of the necessity of vigilance and thorough patient assessment before and after laser treatments to minimize the risk of complications. Furthermore, the authors encourage further studies and case reports to better understand the association between laser treatments and ocular inflammatory conditions.

Gunes et al. [15] expanded on this issue by presenting two additional cases of anterior uveitis following eyebrow epilation. Both patients developed symptoms consistent with anterior uveitis, such as eye redness, pain, sensitivity to light (photophobia), and blurred vision after undergoing the procedure. The onset of symptoms was noted shortly after the laser treatment, which raised concern about a potential causal relationship between the laser epilation and the inflammatory condition. Both cases underwent thorough ophthalmological examination, leading to the diagnosis of anterior uveitis. Standard treatment protocols were initiated, which included the administration of topical corticosteroids and mydriatic agents to manage inflammation and alleviate discomfort. The authors highlighted the potential mechanisms behind this complication, suggesting that thermal injury from the laser, the inflammatory response to the treatment, or even the possibility of pigment dispersion could lead to inflammation of the uveal tract. They emphasized the proximity of the treatment area to the eyes and the need for practitioners to be aware of such ocular complications that may arise from seemingly peripheral cosmetic procedures. The report serves as a cautionary note for ophthalmologists and dermatologists about the rare yet possible occurrence of anterior uveitis following laser treatments in areas near the eyes. The authors recommended careful patient evaluation and counseling about the potential risks associated with laser procedures, even when the treatment is conducted at a distance from the ocular region.

### 3.3. Risks from Intense Pulsed Light and Other Light-Based Treatments

Jewsbury and Morgan [16] investigated the ocular side effects of Intense Pulsed Light (IPL) therapy, focusing on a case of anterior uveitis and iris photoablation. The authors highlighted that while IPL therapy is effective for skin conditions, it can cause significant ocular complications. The patient, who underwent IPL treatment for facial freckles, developed anterior uveitis, pain, and photophobia, leading to diffuse conjunctival injection, intrastromal hemorrhages, and iris transillumination defects. Although the uveitis resolved with treatment, the patient experienced permanent anisocoria and severe glare. This case underscores the potential for severe ocular damage resulting from IPL therapy and emphasizes the necessity for enhanced ocular protection and awareness among practitioners.

Pang and Wells [17] reported a case of bilateral anterior uveitis following IPL therapy for pigmented eyelid lesions. The patient, a 50-year-old dental assistant, experienced ocular discomfort and inflammation after her third IPL session. Clinical examination revealed anterior chamber cytosis, increased iris vascularity, and persistent light adaptation issues due to posterior synechiae. The paper stresses that while IPL is intended for skin rejuvenation, its impact on pigmented tissues like the iris can result in serious adverse effects. The case highlights the need for stringent ocular safety measures and better training for IPL practitioners to prevent similar complications.

Kazak et al. [18] presented a case study of a 24-year-old female who developed photophobia, discoria, and anterior chamber inflammation following IPL therapy for posttraumatic subcutaneous hemorrhage. Despite her best corrected visual acuity being 20/20, she suffered from persistent pupil dilatator dysfunction, anterior chamber cytosis, and increased iris vascularity. The treatment included topical tropicamide and dexamethasone, but there was no restoration of pupil dilatator function at the three-month follow-up. This case demonstrates the potential for persistent ocular damage, such as iris burn and iritis, associated with IPL therapy, highlighting the importance of preventive measures and adequate ocular protection.

Crabb et al. [19] discussed the risk of IPL-induced ocular damage in a case involving a 28-year-old woman who developed iritis and persistent iris atrophy after IPL therapy for facial telangiectasia and capillary vascular malformation. Despite using disposable eye shields, the patient experienced significant anterior chamber inflammation and persistent symptoms, including dyscoria and iris atrophy. The study emphasizes the necessity of optimal ocular protection and informed consent, noting that the highly pigmented iris is particularly susceptible to IPL wavelengths. The authors advocate for stringent safety precautions and awareness of potential ocular risks in IPL treatments.

### 3.4. Complications Arising from Diode and Alexandrite Lasers

Sheikh et al. [20] reported on a significant case involving the development of uveitis and visual field defects following diode laser therapy. This case, categorized as Level IV evidence, illustrates the inflammatory response that can occur following such treatments. The patient developed symptoms such as pain, blurred vision, and photophobia, which, if untreated, could have progressed to more severe complications, including permanent vision loss. The study also noted the presence of visual field defects, which were carefully assessed through perimetry testing. The authors suggested that the thermal energy from the laser might have caused localized injury to ocular tissues, triggering an inflammatory response. They emphasized the importance of preventive measures, particularly the use of protective eyewear for both patients and practitioners, to reduce the risk of accidental exposure to laser energy. The necessity of educating patients and healthcare providers about the potential ocular complications linked to laser treatments was also highlighted, as this knowledge can lead to more informed decision-making and better prevention strategies.

Parver et al. [21] further explored the risks associated with laser hair removal, particularly in the eyebrow region. Their study presented several cases of ocular injuries, including chemical burns, corneal abrasions, and potential retinal damage, all of which underscore the narrow safety margin when performing cosmetic laser treatments near sensitive ocular structures (Level IV). The injuries were primarily attributed to inadvertent laser exposure and inadequate protective measures during the procedures. The authors emphasized the critical need for best practices in this area, advocating for the consistent use of appropriate eye protection for both patients and practitioners. They also stressed the importance of thorough patient evaluation and informed consent, ensuring that individuals are fully aware of the potential complications before undergoing such treatments. This proactive approach can contribute to better patient outcomes and increased safety standards in cosmetic procedures involving lasers.

Lin et al. [22] focused on the adverse effects of alexandrite lasers used in cosmetic procedures around the periorbital area. In their case study, classified as Level IV evidence, they described a patient who developed iritis and pupillary distortion following a laser treatment intended for hair removal near the eyes. These complications, while rare, have serious implications, particularly when lasers are used near sensitive ocular structures. The authors discussed the importance of adhering to stringent safety protocols, including the use of protective eyewear and ensuring that practitioners are well-versed in both laser technology and ocular anatomy to prevent such adverse outcomes. The study concluded with recommendations for increased practitioner training and patient education to mitigate the risks associated with these treatments.

Brilakis et al. [23] provided another case report that detailed significant ocular injuries resulting from diode laser therapy used for hair removal in the periocular region. The patient in this case developed cataracts and iris atrophy after a series of treatments, highlighting the serious risks associated with laser procedures near the eyes (Level IV). The authors suggested that the thermal energy generated by the diode laser likely caused these complications, emphasizing the importance of protective measures, including rigorous training for practitioners and strict adherence to safety protocols. They concluded that while laser hair removal can be an effective cosmetic treatment, it is essential to consider the potential for significant ocular risks, particularly in areas close to critical structures like the eyes.

### 3.5. Broad Overview of Ocular Risks in Dermatologic Laser Treatments

Gulmez et al. [24] addressed the ocular complications that can arise from the cosmetic use of diode lasers in the periocular region, particularly on the eyelids (Level IV). The study presented cases where patients experienced complications such as burns, corneal abrasions, and other types of ocular surface damage following diode laser procedures aimed at cosmetic enhancement.

The authors emphasized the importance of protective measures, including the use of appropriate eye shields and ensuring practitioners have specific training for these procedures. They advocated for more stringent safety protocols and comprehensive training to minimize risks and enhance patient safety. The need for patient education and informed consent was also highlighted, ensuring patients understand the potential risks before undergoing such treatments.

Bonińska et al. [25] conducted an extensive review of ocular and periocular complications arising from dermatologic laser treatments. This study, classified as Level IV evidence, highlighted the increasing popularity of laser procedures in dermatology and the corresponding need for awareness of the potential complications that could affect the eyes. The authors categorized these complications into several groups, including thermal injuries, corneal damage, retinal complications, and issues arising from non-compliance with safety protocols. They emphasized that even minimal laser exposure can lead to severe and irreversible ocular damage, underscoring the critical need for protective measures such as proper eyewear during procedures. Additionally, the study highlighted the necessity for dermatologists to receive thorough training in laser physics and safety protocols to prevent accidents and protect patient vision.

Huang et al. [26] provided an in-depth examination of the risks associated with ocular injuries that may occur during cosmetic laser procedures performed on the facial region. The authors provided a comprehensive overview of the various types of lasers used in these procedures, including fractional lasers, non-ablative lasers, and ablative lasers (Level IV). They analyzed the mechanisms through which these lasers can cause ocular injuries, highlighting specific types of injuries such as thermal burns, corneal abrasions, and retinal damage. The study emphasized that ocular injuries could occur due to direct laser exposure to the eye or from scattered laser light. The authors discussed factors that increase the risk of injury, including the proximity of the laser treatment area to the eyes, the patient’s positioning, and the practitioner’s experience. An important focus of the article was on the preventive measures that practitioners should take to minimize the risk of ocular injury, such as using appropriate eye protection, ensuring proper patient positioning, and adhering to safety protocols during procedures. The authors also advocated for thorough patient education regarding the potential risks involved in cosmetic laser treatments, emphasizing the importance of vigilance among both practitioners and patients.

Flegel et al. [27] provided a comprehensive review of ocular injuries that can occur as a result of dermatological laser treatments, which are increasingly used for cosmetic and medical applications. This review, classified as Level IV evidence, systematically analyzed studies and case reports detailing different types of ocular injuries, categorizing them based on the type of laser and the specific dermatological procedure performed. The authors highlighted a range of ocular complications, including corneal damage, retinal burns, and even permanent vision loss, underscoring the importance of rigorous eye protection during laser therapies. The review also discussed the mechanisms behind these injuries, such as direct laser beam exposure, light reflection, and thermal injury from high-intensity light sources. The authors emphasized the need for meticulous precautions, including the use of appropriate protective eyewear for both patients and practitioners, to prevent such injuries. This comprehensive review serves as a crucial resource for dermatologists and cosmetic practitioners, providing detailed insights into the risks associated with various laser treatments and emphasizing the importance of preventive measures to protect ocular health.

### 3.6. Case Studies Highlighting Severe Ocular Injuries

Artuç et al. [28] provided a detailed account of a case involving retinal injury stemming from a laser hair removal procedure. This report, classified as Level IV evidence, is significant as it highlights the potential ocular risks associated with laser epilation, a common cosmetic treatment that uses concentrated light to remove hair. The patient in this case suffered retinal damage during a laser epilation session targeted at the facial area. The incident underscores the proximity of the treatment zone to sensitive ocular structures like the retina, which are at risk during such procedures. The authors meticulously documented the diagnostic process and the subsequent management of the ocular injury, which included a thorough examination and a discussion of the treatment options available for retinal damage. The case was analyzed to raise awareness about the risks associated with laser treatments, particularly those conducted near the eyes. The authors emphasized the importance of implementing rigorous safety protocols, including the use of protective eyewear for both patients and practitioners, to prevent such incidents. This case serves as a wake-up call for the cosmetic dermatology community, reinforcing the need for vigilance and adherence to safety guidelines during laser epilation.

Tofolean et al. [29] discussed a case of choroidal neovascularization that occurred as a complication following a laser hair removal procedure. This publication, providing Level IV evidence, emphasizes the ocular risks associated with cosmetic laser treatments, particularly those targeting areas close to the eyes. In the case presented, the authors detailed the clinical features observed in a patient who developed choroidal neovascularization after undergoing laser epilation in the facial region. Choroidal neovascularization is characterized by the growth of new, abnormal blood vessels in the choroid layer of the eye, which can lead to serious visual complications. The authors explained that this condition likely developed due to the thermal effects from the laser, which may have triggered the neovascularization process. They highlighted the importance of postoperative monitoring for patients who undergo cosmetic laser procedures, particularly those in close proximity to the eyes. Preventive measures, such as the use of protective eyewear and careful planning of treatment parameters, were strongly advocated to mitigate the risk of ocular complications. This case report serves as a reminder to healthcare providers in the field of esthetic dermatology to remain vigilant about the potential ocular side effects associated with their procedures.

Halkiadakis et al. [30] presented a case report that highlights significant ocular complications, specifically iris atrophy and posterior synechiae, associated with eyebrow laser hair removal. This study, classified as Level IV evidence, emphasizes the risks posed to ocular health when lasers are used near sensitive eye structures, even in routine cosmetic procedures. Iris atrophy, which involves the thinning or degeneration of iris tissue, and posterior synechiae, adhesions that form between the iris and the lens of the eye, are both serious conditions that can lead to complications such as vision problems and increased intraocular pressure. The authors explained that these complications, although rare, can occur when proper precautions are not taken during laser treatments. They discussed the mechanisms that could lead to such ocular injuries, suggesting that thermal or direct laser damage might be responsible. The authors highlighted the importance of using protective measures, such as appropriate eye shields, during laser procedures to safeguard vulnerable ocular structures. They concluded by advocating for increased awareness among dermatologists and laser practitioners regarding the potential for such complications and the necessity of patient education about the possible risks associated with these treatments.

### 3.7. Awareness and Prevention of Ocular Complications

Yan et al. [31] provided a comprehensive review of the various ocular injuries that can arise from a wide range of cosmetic procedures, emphasizing the importance of understanding these risks for both practitioners and patients. This study, classified as Level IV evidence, systematically reviewed documented cases of ocular complications following treatments such as chemical peels, laser treatments, fillers, and other dermatological services. The authors discussed the mechanisms by which these procedures can lead to eye injuries, which may include thermal damage, chemical exposure, and physical trauma. Specific examples of injuries included corneal burns, retinal detachment, and elevated intraocular pressure. The review also examined the implications of these complications, noting that many cosmetic procedures are performed in non-medical settings where practitioners may not have extensive training in recognizing or managing ocular injuries. The authors advocated for proper training and adherence to safety protocols during cosmetic treatments to prevent adverse outcomes. They highlighted the necessity for practitioners to conduct thorough assessments of patients’ ocular health prior to treatments, as well as to provide adequate post-procedure care and education about potential risks. The study emphasized the need for increased awareness among patients regarding the potential ocular risks associated with cosmetic procedures, recommending that practitioners use protective measures such as eye shields or goggles, particularly during procedures performed near the eyes, to minimize the risk of injuries.

Wong et al. [32] provided an in-depth examination of ocular injuries resulting from laser treatments. This narrative review, classified as Level IV evidence, aimed to raise awareness of the potential risks associated with the increasing use of lasers in both medical and cosmetic procedures. The authors discussed various types of lasers used in ophthalmology and cosmetic surgery, including their intended applications and potential mechanisms of injury. They detailed the mechanisms by which laser exposure could lead to ocular injuries, such as thermal damage, photochemical reactions, and mechanical trauma. The review presented a wide range of possible ocular complications, including corneal burns, retinal damage, cataracts, and other vision-threatening injuries. The authors highlighted the incidence of laser-induced ocular injuries and the factors that could increase the risk of such complications. These factors included the type of laser used, the duration and intensity of exposure, and the proximity of the laser beam to the eye. Furthermore, the review emphasized that many of these injuries could occur not only during medical procedures but also during cosmetic applications, where practitioners might not have adequate training in managing ocular safety. The authors concluded by emphasizing the importance of preventive measures, such as employing appropriate protective eyewear for both patients and operators during laser procedures and advocated for improved training for practitioners to ensure a thorough understanding of laser safety and ocular anatomy. They also called for further research and reporting of cases involving laser-induced ocular injuries to better understand the long-term outcomes of such injuries and to develop standardized protocols for preventing and managing these complications.

Collea et al. [33] presented a clinical case discussing the risks of ocular complications that can arise from cosmetic laser procedures, specifically focusing on eyebrow photothermolysis. This publication, classified as Level IV evidence, provided a brief overview of the use of laser technology for esthetic treatments, emphasizing the potential complications related to the proximity of treatment areas to the eyes. The report documented specific cases where patients experienced complications such as thermal injuries and pigmentary changes following bilateral eyebrow laser treatment. The authors stressed the importance of understanding facial anatomy and using appropriate safety measures to minimize risks during such procedures. Furthermore, they highlighted the need for patient education regarding the risks of laser treatments, advocating for thorough pre-treatment consultations to inform patients of potential complications. The discussion added context to the cases presented, contributing to the growing body of literature on the safety and efficacy of cosmetic laser procedures. The authors concluded by advocating for ongoing research into the long-term effects and outcomes associated with such complications, as well as the development of standardized guidelines to improve safety protocols in clinical settings.

Shulman et al. [34] addressed the potential risks to ocular health associated with laser-assisted hair removal, particularly during eyebrow epilation. This study, classified as Level IV evidence, detailed cases where patients experienced ocular complications such as thermal injury and corneal damage following laser procedures. The authors emphasized that while laser-assisted hair removal, including eyebrow epilation, is a popular cosmetic procedure, it is not without risks, particularly concerning the eyes. The study highlighted the anatomical proximity of the treatment area to the eyes, stressing the importance of implementing safety measures to protect patients during the procedure. Protective eyewear was emphasized as a critical component in minimizing the risk of ocular injury, along with proper technique and equipment calibration. The authors advocated for increased awareness among practitioners performing laser epilation about the potential for such complications, calling for careful patient selection and thorough pre-treatment consultations to inform patients about the risks involved. Overall, the article served as a reminder of the importance of ocular safety in esthetic procedures involving laser technology, contributing to the ongoing discussion about patient safety and the need for enhanced protocols in laser-assisted cosmetic treatments.

The reviewed studies provide comprehensive insights into the various ocular complications that can arise from laser- and light-based cosmetic treatments. These complications, ranging from corneal abrasions to severe retinal damage, underscore the critical need for stringent safety protocols and thorough practitioner training. Table 1 summarizes the key findings, types of ocular injuries, and recommended preventive measures from each study, highlighting the importance of vigilance and patient education in minimizing risks associated with these procedures.

**Table 1:** This table summarizes the ocular complications and key findings from reviewed studies on laser- and light-based treatments. It provides a structured overview of the significant ocular risks, complications, and recommendations associated with various cosmetic treatments using lasers and light-based technologies.

Reference	Study Type (Level of Evidence)	Laser Type/Treatment	Ocular Complications	Key Findings/Recommendations
Park et al. [10]	Case Report (Level IV)	Neodymium-doped Yttrium Aluminum Garnet Laser (Nd)	Severe macular injury	Emphasized the critical need for rigorous safety protocols, proper equipment handling, and thorough training of practitioners to prevent significant visual impairment.
Chen et al. [11]	Case Report (Level IV)	Cosmetic Laser for Skin Rejuvenation	Retinal injury	Highlighted the necessity for stringent safety protocols and adequate practitioner training, especially in non-medical settings, to prevent retinal damage during cosmetic procedures.
Widder et al. [12]	Case Report (Level IV)	Carbon Dioxide (CO_2_) Laser Skin Resurfacing	Corneal damage (superficial punctate keratitis, corneal opacity)	Stressed the importance of immediate identification and treatment of corneal damage and recommended the use of protective eye gear during laser resurfacing procedures.
Hammes et al. [13]	Case Report (Level IV)	Laser Treatment for Port-Wine Stain	Pupillary dysfunction	Emphasized the need for understanding the anatomical proximity of ocular structures when performing laser procedures and advocated for the use of protective measures like eye shields.
Karabela et al. [14]	Case Report (Level IV)	Alexandrite Laser for Eyebrow Epilation	Anterior uveitis	Underlined the importance of pre-treatment information and careful patient assessment before and after laser treatments near sensitive areas like the eyes.
Gunes et al. [15]	Case Series (Level IV)	Alexandrite Laser for Eyebrow Epilation	Anterior uveitis	Highlighted the potential mechanisms behind uveitis development and emphasized the need for vigilance among practitioners and the importance of patient education.
Jewsbury and Morgan [16]	Case Report (Level IV)	Intense Pulsed Light (IPL) Therapy	Anterior uveitis, Iris photoablation	Highlighted significant ocular complications, emphasizing the necessity for enhanced ocular protection and awareness among practitioners during IPL therapy.
Pang and Wells [17]	Case Report (Level IV)	Intense Pulsed Light (IPL) Therapy	Bilateral anterior uveitis	Stressed the need for stringent ocular safety measures and better training for IPL practitioners to prevent serious adverse effects like bilateral anterior uveitis.
Kazak et al. [18]	Case Study (Level IV)	Intense Pulsed Light (IPL) Therapy	Photophobia, discoria, anterior chamber inflammation	Demonstrated the potential for persistent ocular damage, such as iris burn and iritis, associated with IPL therapy, highlighting the importance of preventive measures and adequate ocular protection.
Crabb et al. [19]	Case Report (Level IV)	Intense Pulsed Light (IPL) Therapy	Iritis, persistent iris atrophy	Emphasized the necessity of optimal ocular protection and informed consent, noting the susceptibility of the iris to IPL wavelengths.
Sheikh et al. [20]	Case Report (Level IV)	Diode Laser Therapy	Uveitis, visual field defects	Highlighted the inflammatory response and the importance of preventive measures and patient education to reduce the risk of permanent vision loss.
Parver et al. [21]	Case Series (Level IV)	Laser Hair Removal (Eyebrow Region)	Chemical burns, corneal abrasions, retinal damage	Emphasized the critical need for best practices, including appropriate eye protection and thorough patient evaluation, to prevent ocular injuries during cosmetic procedures.
Lin et al. [22]	Case Report (Level IV)	Alexandrite Laser (Periorbital Area)	Iritis, pupillary distortion	Discussed the importance of stringent safety protocols and recommended increased practitioner training to prevent adverse outcomes in sensitive ocular regions.
Brilakis et al. [23]	Case Report (Level IV)	Diode Laser Therapy (Periocular Region)	Cataracts, iris atrophy	Highlighted the risks associated with diode laser therapy near the eyes, emphasizing the importance of protective measures and strict adherence to safety protocols.
Gulmez et al. [24]	Case Series (Level IV)	Diode Laser (Eyelids)	Burns, corneal abrasions	Advocated for protective measures and comprehensive training to minimize risks in cosmetic procedures involving diode lasers near the eyes.
Bonińska et al. [25]	Review (Level IV)	Various Dermatologic Laser Treatments	Thermal injuries, corneal damage, retinal complications	Emphasized the need for protective measures and proper training to prevent severe ocular damage during dermatologic laser treatments.
Huang et al. [26]	Review (Level IV)	Various Cosmetic Lasers	Thermal burns, corneal abrasions, retinal damage	Highlighted the importance of preventive measures, proper patient positioning, and thorough practitioner training to minimize the risk of ocular injuries during cosmetic procedures.
Flegel et al. [27]	Review (Level IV)	Dermatological Laser Treatments	Corneal damage, retinal burns, permanent vision loss	Stressed the need for meticulous precautions and the use of appropriate protective eyewear during laser therapies to prevent severe ocular injuries.
Artuç et al. [28]	Case Report (Level IV)	Laser Hair Removal	Retinal injury	Emphasized the importance of rigorous safety protocols, including the use of protective eyewear, to prevent ocular injuries during laser epilation near the face.
Tofolean et al. [29]	Case Report (Level IV)	Laser Hair Removal	Choroidal neovascularization	Highlighted the importance of postoperative monitoring and preventive measures, including the use of protective eyewear, to mitigate ocular risks in laser procedures.
Halkiadakis et al. [30]	Case Report (Level IV)	Eyebrow Laser Hair Removal	Iris atrophy, posterior synechiae	Advocated for the use of protective measures and patient education to prevent complications during laser treatments near the eyes, even in routine cosmetic procedures.
Yan et al. [31]	Review (Level IV)	Various Cosmetic Procedures	Corneal burns, retinal detachment, elevated intraocular pressure	Discussed the importance of proper training, safety protocols, and patient education to prevent ocular complications associated with cosmetic procedures.
Wong et al. [32]	Review (Level IV)	Various Laser Treatments	Corneal burns, retinal damage, cataracts	Emphasized the importance of preventive measures, proper technique, and patient education to mitigate the risks of laser-induced ocular injuries.
Collea et al. [33]	Case Report (Level IV)	Eyebrow Photothermolysis	Thermal injuries, pigmentary changes	Highlighted the significance of understanding facial anatomy and using appropriate safety measures to minimize risks during cosmetic laser procedures.
Shulman et al. [34]	Case Series (Level IV)	Laser-Assisted Hair Removal	Thermal injury, corneal damage	Stressed the importance of protective eyewear, proper technique, and thorough patient education to minimize the risk of ocular injuries during laser hair removal treatments.

## 4. Discussion

The use of laser and light therapies in medical and esthetic practices has become widespread due to their precision and efficacy in treating various skin conditions. The fundamental principle behind these treatments is selective photothermolysis, which enables the destruction of targeted tissues, such as blood vessels, while sparing the surrounding structures [35]. For successful selective photothermolysis, three critical conditions must be met: (1) a wavelength that penetrates deeply enough to be preferentially absorbed by the target chromophore, (2) an exposure duration that matches the target’s thermal relaxation time (TRT), and (3) sufficient energy (fluence) to cause permanent damage to the target. This mechanism is particularly effective in treating vascular lesions, where thermal injury to oxyhemoglobin leads to coagulation, perivascular collagen damage, and vessel wall necrosis, with minimal damage to the surrounding tissues [36,37].

However, the same characteristics that make lasers effective in treating skin lesions—such as coherence and low divergence—can also cause serious ocular damage if safety precautions are not strictly followed. The eye, especially the retina, is highly susceptible to laser-induced damage due to its optical focusing system, which can concentrate laser energy to dangerous levels [38].

Lasers have the potential to inflict a wide range of ocular injuries, depending on the specific structure of the eye involved. For instance, the cornea, which contains a high density of pain receptors, can experience severe pain even from minor thermal injuries [39]. Although corneal damage limited to epithelium typically does not result in significant vision impairment [40], damage to the retina or lens can lead to serious visual consequences. Retinal damage is particularly concerning because the retina is responsible for converting light into visual signals for the brain. The lens of the eye focuses light from the visible to near-infrared spectrum (400–1400 nm) onto the retina, producing retinal irradiance that is 105 times greater than corneal irradiance [41]. Therefore, extreme caution is required when using cosmetic lasers, particularly those applied to the upper face, as many dermatological lasers fall within this hazardous wavelength range.

Eye injuries represent a significant risk associated with laser treatments. The use of lasers within the visible spectrum (400–1400 nm) increases the likelihood of retinal injury. Table 2 provides an overview of the common ocular complications, their causes, and the associated laser or light-based treatments. Lasers operating in the infrared (200–400 nm) and ultraviolet (1400–10,600 nm) ranges primarily cause minor damage to the cornea and/or lens. Factors that exacerbate the severity of ocular damage include pupil dilation, foveal involvement, higher fluence combined with brief laser pulses, and the patient’s retinal pigmentation [42].

The symptoms of laser-induced eye damage vary depending on the type of laser used and can include sudden blindness, photophobia (which may be permanent), pain (more common with Erbium: YAG or CO_2_ lasers), oval pupil, conjunctival synechiae, difficulty in color recognition due to retinal damage, and floaters, which are frequently observed after pulsed dye laser treatments [43].

Preventing eye injuries during laser procedures is critical and can be achieved through the use of appropriate protective eyewear or eye coverings. It is essential to fully shield the iris, and for contact lens wearers, the application of ophthalmic ointment and anesthetic eye drops (such as oxybuprocaine and tetracaine) can aid in re-epithelialization. Attention to detail is required to ensure that glasses or lenses remain correctly positioned throughout the procedure [44]. In the event of an injury, urgent evaluation by an ophthalmologist is necessary to determine the appropriate treatment, which will vary based on the type of lesion and laser used [45].

The tissue’s affinity for a laser’s wavelength determines its absorption, with specific chromophores in the tissue preferentially absorbing certain wavelengths. Melanin, hemoglobin, and water are the three primary organic chromophores of therapeutic significance. For instance, oxyhemoglobin, the predominant form of hemoglobin, is targeted to treat vascular lesions, with absorption peaks at 418, 542, and 577 nm [46]. Wavelength also affects scattering, with longer wavelengths leading to less tissue penetration and more scattering compared to shorter wavelengths. Additionally, factors such as power (watts/s), spot size (cm^2^), and exposure duration influence the performance of lasers. Larger spot sizes result in deeper tissue penetration but reduced scattering [47].

Despite the precision of laser targeting, collateral damage can occur due to surrounding scatter and the resultant heat effects. Thermal damage ensues when a chromophore absorbs energy at a rate faster than it can dissipate the generated heat. While the primary focus is on tissue chromophores, other ocular structures rich in these chromophores—such as the retina (rich in melanin and hemoglobin), uvea (rich in melanin), and cornea and lens (rich in water)—can also be inadvertently harmed [48].

Different mechanisms can lead to laser-induced ocular damage, depending on the type of laser and the characteristics of the specific ocular structures involved. The crystalline lens’s focusing ability makes lasers in the retinal hazard region (400–1400 nm) particularly dangerous, with the potential to cause severe macular damage. For example, the argon laser’s wavelengths (488 nm and 515 nm) are primarily absorbed by melanin and blood vessels, increasing the risk of retinal damage [49]. Improper use of eye protection can allow the laser to penetrate the cornea, focus onto the retina through the lens, and cause photocoagulation by heat denaturation of proteins [50].

Near- and mid-infrared lasers, such as Nd lasers (1064 nm) used for vascular lesions, hair removal, and rejuvenation, are less attracted to melanin, but they can still cause retinal damage [51]. Notably, lasers operating in the 700–1400 nm range pose a particular threat because they are invisible to the human eye yet capable of causing significant ocular harm.

The Erbium: Yttrium aluminum garnet laser, operating at a wavelength of 2940 nm, is a fractionally applied ablative laser that is often preferred over the carbon dioxide laser due to its reduced thermal damage. This advantage stems from the Erbium laser’s superior absorption by water and collagen, resulting in more precise tissue ablation with minimal thermal spread. Nevertheless, the use of this laser is not without risks, as it has been associated with complications such as erythema, hyperpigmentation, hypopigmentation, skin infections, and unintentional ocular injury [52]

IPL technology, although non-ablative, is extensively employed for the treatment of skin pigmentation disorders, irregular skin texture, and telangiectasia. Unlike lasers, IPL devices emit a broad spectrum of non-collimated, non-coherent light, typically within the 500 to 1200 nm range, which unfortunately falls within the retinal hazard zone. Despite not being categorized as a laser, IPL poses significant ocular risks, particularly to pigmented structures such as the iris, which are susceptible to accidental damage during treatments [53]. The literature documents severe adverse effects following IPL therapy, including intense ocular pain, anterior uveitis, pupillary distortion, persistent iris atrophy, and photophobia [16,17,18,19], as well as bilateral anterior uveitis after Intense Pulsed Light therapy for pigmented eyelid lesions [54,55]. A common issue with light-based therapies is the misplaced sense of safety; many practitioners may underestimate the potential for ocular damage. Most injuries are attributed to inadequate eye protection or the improper adjustment of eye shields, especially when treating challenging areas such as the medial canthus. It is imperative to acknowledge and address the risks associated with both laser- and light-based treatments to ensure the safety and well-being of patients.

## 5. Conclusions

Laser- and light-based therapies are invaluable tools in cosmetic facial procedures; however, their application near the periorbital region or on the eyelids carries a significant risk of ocular injury. To mitigate these risks, several precautionary measures are essential. First, it is crucial that the attending physician possesses a thorough understanding of laser physics and safety protocols, coupled with specialized training in the operation of these devices. Second, lesions located near the eyes or on the eyelids should never be treated without the use of appropriate protective measures, such as metal ocular shields or wavelength-specific protective glasses, which also safeguard the operating staff. During lengthy procedures, meticulous care must be taken to ensure that eye protection remains securely in place and does not become dislodged.

Furthermore, to prevent thermal injury and avoid overheating of metal corneal shields, consistent and adequate cooling of the treated areas is imperative. It is also strongly recommended that laser treatments in the periorbital or eyelid regions be performed exclusively by core physicians, such as board-certified dermatologists, plastic surgeons, ophthalmologists, or otolaryngologists with extensive training in cosmetic surgery and medicine. Despite the implementation of these stringent safety protocols, the potential for laser-induced ocular damage, though rare, still exists. In cases of superficial corneal injury, treatment options may include the application of topical steroids, the use of therapeutic contact lenses, or the placement of protective eye patches to facilitate healing.

## Figures and Tables

**Table 2:** Summary of ocular complications and key findings from reviewed studies on laser- and light-based treatments. This table provides a structured overview of the key findings, complications, and recommendations from the reviewed studies, highlighting the significant ocular risks associated with various laser- and light-based cosmetic treatments.

Ocular Complication	Cause/Mechanism	Associated Laser/Light Treatment
Macular Injury	Misalignment of laser system, improper focusing of laser energy	Nd
Laser		
Retinal Damage	Unintended laser beam exposure, improper eye protection, thermal injury	Cosmetic Lasers (e.g., skin rejuvenation), Argon Laser, Nd
Laser		
Corneal Damage	Direct laser exposure, inadequate protective measures, thermal injury	CO_2_ Laser Skin Resurfacing, Cosmetic Lasers, IPL
Pupillary Dysfunction	Collateral damage to ocular structures due to proximity of laser treatment	Laser treatment for facial port-wine stain
Anterior Uveitis	Thermal injury, inflammatory response, pigment dispersion	Alexandrite Laser for Eyebrow Epilation, IPL
Iritis and Pupillary Distortion	Inflammatory response, improper eye protection	Alexandrite Laser (Periorbital Area), IPL
Cataracts	Thermal or mechanical injury to the lens, photocoagulation	Diode Laser Therapy, IPL
Corectopia	Mechanical or thermal injury leading to abnormal pupil displacement	Laser Eyebrow Epilation
Photophobia and Persistent Iris Atrophy	Chronic inflammation, thermal injury to iris	IPL, Erbium
Laser		
Choroidal Neovascularization	Thermal effects from laser triggering neovascularization	Laser Hair Removal
Conjunctival Synechiae	Inflammation, improper use of protective measures during laser treatment	CO_2_ Laser, Erbium
Laser		
Floaters	Photochemical reactions, thermal damage to the retina	Pulsed Dye Laser, other dermatological lasers
